# Exposure to selenomethionine causes selenocysteine misincorporation and protein aggregation in *Saccharomyces cerevisiae*

**DOI:** 10.1038/srep44761

**Published:** 2017-03-17

**Authors:** Pierre Plateau, Cosmin Saveanu, Roxane Lestini, Marc Dauplais, Laurence Decourty, Alain Jacquier, Sylvain Blanquet, Myriam Lazard

**Affiliations:** 1 Laboratoire de Biochimie, Ecole Polytechnique, CNRS UMR7654, 91128 Palaiseau Cedex, France; 2 Institut Pasteur, Unité de Génétique des Interactions Macromoléculaires, CNRS-UMR3525, Paris, France; 3 Laboratoire d’Optique et Biosciences, Ecole Polytechnique, CNRS UMR7645-INSERM U1182 91128, Palaiseau Cedex, France

## Abstract

Selenomethionine, a dietary supplement with beneficial health effects, becomes toxic if taken in excess. To gain insight into the mechanisms of action of selenomethionine, we screened a collection of ≈5900 *Saccharomyces cerevisiae* mutants for sensitivity or resistance to growth-limiting amounts of the compound. Genes involved in protein degradation and synthesis were enriched in the obtained datasets, suggesting that selenomethionine causes a proteotoxic stress. We demonstrate that selenomethionine induces an accumulation of protein aggregates by a mechanism that requires *de novo* protein synthesis. Reduction of translation rates was accompanied by a decrease of protein aggregation and of selenomethionine toxicity. Protein aggregation was supressed in a ∆*cys3* mutant unable to synthetize selenocysteine, suggesting that aggregation results from the metabolization of selenomethionine to selenocysteine followed by translational incorporation in the place of cysteine. In support of this mechanism, we were able to detect random substitutions of cysteinyl residues by selenocysteine in a reporter protein. Our results reveal a novel mechanism of toxicity that may have implications in higher eukaryotes.

Selenium is an essential micronutrient for many living species, including humans. It is translationally incorporated as selenocysteine (SeCys) into a few proteins, some of which are antioxidant enzymes, protecting cells from harmful oxidative damage^1^. Incorporation of SeCys occurs via a specific mechanism that recodes a UGA codon from its normal translation termination function^2^. However, at higher doses, many selenium compounds act as pro-oxidants, generating reactive oxygen species (ROS) that induce cytotoxicity and cellular apoptosis^3^. Since the discovery of the first selenoprotein glutathione peroxidase, in 1973^4^, selenium has attracted considerable scientific interest, especially with respect to its potential use as a nutritional supplement in the prevention and treatment of several diseases, including cancer^5^,^6^. In spite of extensive studies the mechanisms of action and cellular targets of selenium compounds are still unclear. As adverse health effects have recently been associated with excessive dietary selenium supplementation, a comprehensive understanding of the molecular basis of selenium toxicity is becoming an important public health issue^7^,^8^,^9^.

Selenium effects depend on its chemical speciation^10^. For example, selenolates (RSe^-^) are redox active compounds that have cytotoxic pro-oxidant properties^3^. Seleno-amino acids such as selenomethionine (SeMet) and selenocysteine^11^,^12^ can be translationally misincorporated in proteins in place of methionine and cysteine, respectively, leading to abnormal and potentially toxic products. Thus, the pathways underlying the biological activity of the different seleno-compounds can be very different and a better understanding of their modes of action is necessary.

Hydrogen selenide, produced by the reduction of inorganic selenium salts (selenate, selenite), is believed to exert a key role in the toxicity as well as in the anticarcinogenic properties of selenium^13^. It reacts with dioxygen and thiols, resulting in the generation of ROS that induce oxidative stress, DNA damage and, ultimately, cell death^14^. Genomic studies using *Saccharomyces cerevisiae* have highlighted the importance of DNA repair pathways in protecting yeast cells against inorganic selenium toxicity. Indeed, many mutants of genes involved in homologous recombination and DNA damage checkpoint showed hypersensitivity to selenide^15^ or selenite^16^,^17^ suggesting that DNA double-strand breaks are a dominant cause of toxicity.

Large-scale approaches in *S. cerevisiae* were also used to study SeMet toxicity. Seitomer *et al*.^16^ showed that tolerance to SeMet treatment was largely unaffected by the loss of most of the genes involved in DNA damage and oxidative stress pathways, suggesting that SeMet toxicity involves mechanisms distinct from those of inorganic selenium. Bockhorn *et al*.^18^ showed that deletion of the *CYS3* gene encoding cystathionine γ-lyase resulted in increased resistance to SeMet. We recently showed that several mutants hypersensitive to H_2_Se display wild-type sensitivity to SeMet, and that *SOD1* deletion imparts sensitivity against SeMet but not against selenide^19^. Furthermore, we found that SeMet toxicity was mediated by the transsulfuration pathway amino acids selenohomocysteine and/or selenocysteine, with the involvement of superoxide production. However, the origin of the toxicity of these metabolites remained unknown. To address this question, we have screened the systematic collection of *S. cerevisiae* haploid knockout strains, previously used to determine biological processes involved in sensitivity to sodium selenide^15^, to analyze the effects of loss of function on growth in the presence of SeMet. This analysis showed the importance of protein degradation pathways to protect cells against SeMet damages. Mass spectrometry analysis and fluorescence microscopy revealed SeCys incorporation in a reporter polypeptide and protein aggregation in yeast cells exposed to SeMet. Our results suggest that SeMet toxicity results from its metabolization into SeCys followed by random incorporation in the place of cysteine, which in turn promotes protein aggregation.

## Results

### Identification of deletion mutants involved in sensitivity or resistance to SeMet

To identify cellular targets of SeMet in *S. cerevisiae*, we screened a collection of ≈4900 isogenic haploid non-essential deletion mutants and ≈1000 haploid DAmP (Decreased Abundance by mRNA Perturbation) mutants of essential genes, by growth in liquid culture of pooled bar-coded mutants. Yeast cells were grown for 10 generations in Synthetic Complete (SC) medium supplemented with 100 μM L-methionine and L-cysteine, in the absence or presence of L-SeMet. SeMet was added at concentrations (12 and 20 μM) that decreased the growth rate of the parental strain (BY4741) by 15 and 25%, respectively. The tags were amplified, and hybridized to barcode specific Agilent microarrays. The hybridization data were used to calculate relative growth values (relative fitness score) for each mutant in the population. The results from three independent experiments and the average value of these results are presented in Supplementary Table S1. As shown in Supplementary Figure 1, the average value obtained for the two SeMet concentrations were highly correlated (r = 0.88). Therefore, only the 20 μM SeMet dataset was used for further analysis. Supplementary Figure 2 shows the distribution of relative fitness scores for each mutant in this dataset.

**Figure 1:**
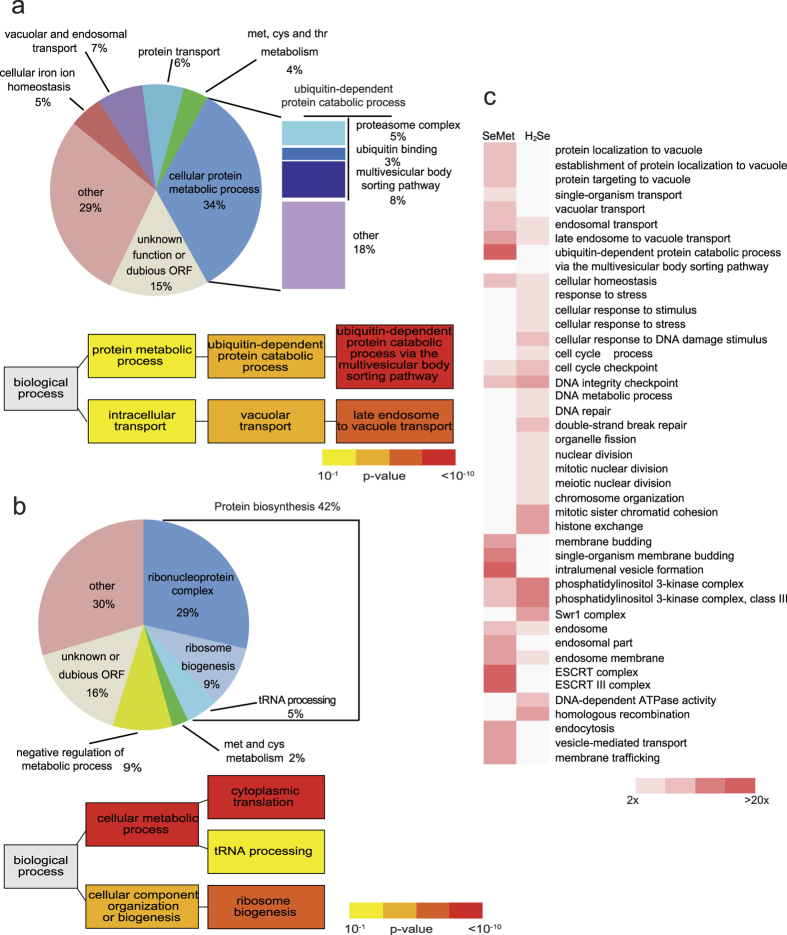
GO term analysis of the SeMet-sensitive and -resistant datasets. (**a**) Distribution of SeMet-sensitive mutants according to biological processes affected (upper panel). Hierarchical graph of GO terms enrichment relative to the genome (lower panel). (**b**) Distribution of SeMet-resistant mutants according to biological processes affected (upper panel). Hierarchical graph of GO terms enrichment relative to the genome (lower panel). The color indicates the p-value of the enrichment according to g:Profiler (yellow: 10^−1^–10^−3^, orange: 10^−3^–10^−6^, light red: 10^−6^–10^−10^, dark red: <10^−10^). (**c**) Functional categories significantly enriched (p < 0.001, fold enrichment >2) in the SeMet- or H_2_Se-sentitive datasets. Only genes for which fitness scores were available in both screens were taken in consideration (137 and 135 genes in the SeMet and H_2_Se datasets, respectively). The color indicates the fold enrichment for each category. Blank boxes indicates an enrichment <2.

A total of 157 mutants (out of 5241 for which at least 2 independent results were obtained, 3%) displayed a growth defect in the presence of SeMet associated to a relative fitness score lower than −1.5 and were defined as SeMet-sensitive strains. The 283 mutants that had a fitness score >1.5 were classified as SeMet-resistant strains. g:Profiler (biit.cs.ut.ee/gprofiler) was used to associate Gene Ontology (GO) terms with the sensitive and resistant datasets (Fig. 1). GO terms related to protein metabolic processes were significantly enriched in both datasets (see Fig. 1a and b and Table 1 for significant genes). Genes related to ubiquitin-mediated protein degradation, either via the proteasome complex or via the multivesicular body sorting pathway, were over-represented among deletion mutants sensitive to SeMet (Fig. 1a). Notably, the six essential ATPases of the proteasome regulatory particle (*RPT1* to *RPT6* genes), necessary for unfolding of cytoplasmic proteins targeted for degradation, are among the most sensitive DAmP mutants. 10 out of the 13 genes coding for the ESCRT complexes, which are involved in recognition and vacuolar targeting of misfolded proteins destined for proteolysis^20^, are present in the sensitive dataset. In addition, this set also contains three subunits of the co-chaperone prefoldin complex involved in the folding of non-native polypeptides^21^.

At the other end of the scale, mutants impaired in the translational process, including ribosomal subunits, proteins necessary for ribosome biogenesis and several tRNA-modifying enzymes represent around 50% of the SeMet-resistant dataset (see Fig. 1b and Table 1 for significant genes). This set also contains several subunits of the Lsm-Pat1 complex, involved in the regulation of mRNA turnover, and transcription factors, among which subunits of the RNA polymerase II mediator and SAGA complexes. These results indicate that SeMet tolerance involves mechanisms related to the folding or removal of damaged proteins, and that resistance to SeMet can be improved by slowing down protein biosynthesis. Both higher sensitivity and resistance of mutant strains point to protein homeostasis as a major actor in SeMet toxicity.

As expected from previous studies, genes involved in sulfur metabolism were found in both datasets (Table 1). In particular, our screen confirmed the resistant phenotype already observed for mutants of *S*-adenosylmethionine synthases (*sam1, sam2*)^22^, methionine permease (*mup1*)^23^ and cystathionine γ-lyase (*cys3*)^18^. A role of superoxide radicals in SeMet toxicity was also confirmed by the sensitive phenotype of the *sod1* and *sod2* mutants^19^.

To determine whether SeMet and H_2_Se toxicity share similar mechanisms, we searched for functional categories that were significantly enriched (p-value < 0.001) in the SeMet- (this study) and the H_2_Se-sensitive subsets^15^. As shown in Fig. 1c, there was little overlap between biological processes required for SeMet and H_2_Se tolerance. This analysis strengthens the idea that the mechanistic bases of selenium effects depend on the specific chemical form.

### Individual validation of selected mutants

The genomic screen was performed in the BY4741 strain with a mutation in *MET17*, the gene that encodes *O*-acetyl homoserine sulfhydrylase, the enzyme responsible for incorporating inorganic sulfur into the amino acid homocysteine. To ascertain that this mutation did not alter SeMet effects, several sensitive or resistant strains, chosen to represent the different pathways affected, were selected for individual analysis in the BY4742 background in which the sulfur assimilation pathway is functional. Genes deleted in the sensitive mutants *ubp6, ubp14* and *mub1* are involved in protein degradation; *LEU3* codes for a transcription factor that regulates genes involved in leucine biosynthesis; *TSA1* and *SOD1* are involved in redox homeostasis; *SRN2, VPS36* and *DID4* code for components of the ESCRT complexes involved in the sorting of proteins into the endosome. Genes deleted in the resistant mutants *uba4* and *urm1* are involved in tRNA modifications and *RPL26B* and *RPL29* code for ribosomal proteins. We measured growth rates of individual strains in the absence or presence of 20 μM SeMet and calculated fitness scores relative to the parental strain. Supplementary Figure 3 shows the average fitness score of individual strains grown in complete medium supporting fast growth (SC + methionine medium) or in a medium in which only auxotrophic requirements are supplied (SD + methionine medium) resulting in a reduced growth rate. In complete medium, all the mutants displayed the phenotype already observed in the genomic screen. This analysis also showed that the growth of several deletion strains, in particular the mutants of ESCRT I and II complexes and ribosomal proteins, was only weakly affected in SD medium, indicating that the effects of SeMet were more pronounced in rapidly growing cells.

### Exposure to SeMet causes protein aggregation

**Figure 2:**
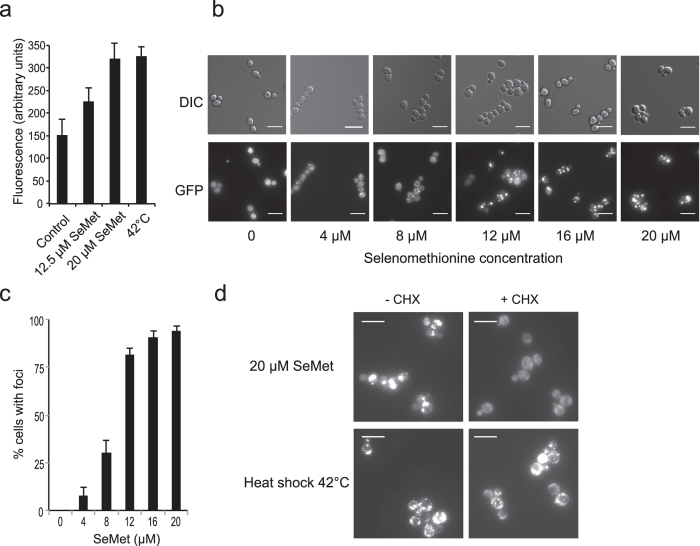
SeMet promotes protein aggregation *in vivo.* (**a**) Induction of Hsp104-GFP by SeMet or heat shock. Exponentially growing BY4741-Hsp104-GFP cells were incubated in SC + 100 μM methionine for 2 h, either at 30 °C in the presence of the indicated concentration of SeMet or at 42 °C in the absence of SeMet. The fluorescence in whole cell extracts was recorded at 508 nm and normalized to the optical density of the extracts at 280 nm. The fluorescence of an extract from untagged BY4741 strain grown in SC + 100 μM methionine was subtracted from the results. The results are the mean ± S. D. of at least 3 experiments. (**b**) Hsp104-GFP localization was monitored by fluorescence microscopy (GFP) and differential interference contrast (DIC), in living BY4741-Hsp104-GFP cells after 1 h of exposure to various SeMet concentrations in SC + 100 μM methionine. Representative images obtained after maximum intensity z-projection, bar equals 10 μm. (**c**) Quantification of protein aggregation. The fraction of cells containing at least one Hsp104-GFP focus was determined by visual inspection of 300–600 cells in each condition. The results are the mean and range of at least 2 experiments. (**d**) Effect of cycloheximide (CHX) on protein aggregation induced by 1 h of exposure to 20 μM SeMet or heat shock at 42 °C. BY4741-Hsp104-GFP cells grown in SC + 100 μM methionine were treated or not with 5 μg/ml cycloheximide for 1 h prior to the SeMet or heat-shock stress. Hsp104-GFP localization was monitored in living cells by fluorescence microscopy. Representative images obtained after maximum intensity z-projection, bar equals 10 μm.

Because genes involved in protein synthesis or degradation featured prominently in the set of sensitive and resistant mutants, we suspected that SeMet induced a proteotoxic stress. To confirm this hypothesis, we used a chromosomally integrated Hsp104-GFP reporter construct. Hsp104p is a chaperone that acts on aggregated proteins and promotes disaggregation and refolding^24^. Upon heat shock, Hsp104p is induced and accumulates at the periphery of protein aggregates, which can thus be visualized as intense cytosolic fluorescent Hsp104-GFP containing foci. First, we questioned the capacity of SeMet to induce a heat-shock-like response and found that the expression of Hsp104-GFP, measured by fluorescence in crude extracts, was induced to similar levels upon exposure to 20 μM SeMet for 2 h or after a temperature shift to 42 °C (Fig. 2a). Then, the localization of Hsp104-GFP was monitored by fluorescence microscopy in cells exposed to increasing concentrations of SeMet (Fig. 2b). Hsp104-GFP distribution was diffuse in the cytoplasm of unexposed cells, whereas concentration-dependent aggregates formed in cells exposed to SeMet (Fig. 2c). At 12 μM SeMet, 80% of cells contained at least one distinct bright fluorescent focus. To determine whether SeMet-triggered protein aggregation required *de novo* protein synthesis, we treated cells with cycloheximide (5 μg/mL), to inhibit translation initiation, prior to the SeMet stress. As shown in Fig. 2d, cycloheximide prevented SeMet-induced aggregate formation, whereas it had little effect on the aggregation induced by a shift to 42 °C, a condition that is known to induce thermal unfolding of native proteins. These results suggest that proteins in the process of synthesis/folding are likely to be prime targets of SeMet-induced aggregation.

**Figure 3:**
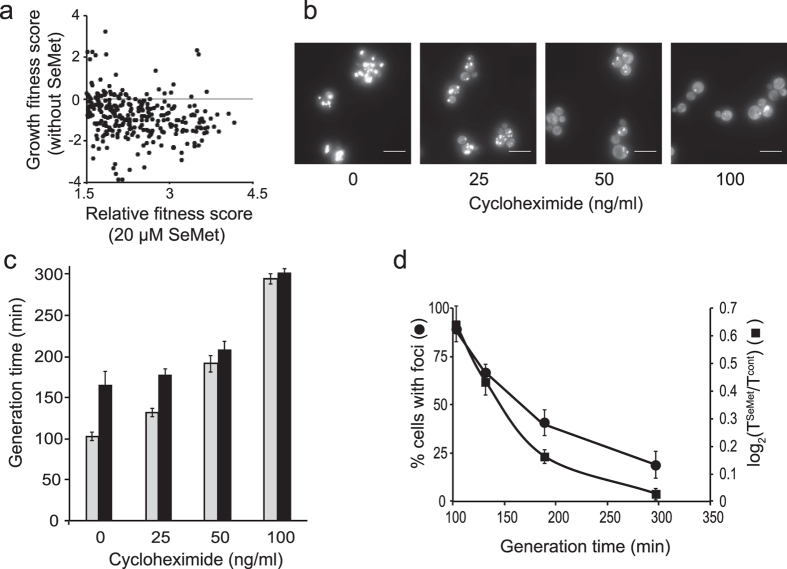
Reduced growth rates affect SeMet toxicity and protein aggregation. (**a**) Relationship between SeMet resistance and growth in the absence of SeMet. Dots correspond to the growth fitness score in the absence of SeMet versus the relative fitness score in the presence of 20 μM SeMet, for strains resistant to SeMet (fitness score >1.5). (**b**) Effect of low cycloheximide concentrations on protein aggregation. Cycloheximide at the indicated concentrations was added to BY4741-Hsp104-GFP cells grown in SC + 100 μM methionine. After 1 h of incubation, 20 μM SeMet was added in the cultures and incubation was continued for 1 h. Hsp104-GFP localization was monitored in living cells by fluorescence microscopy. Representative images obtained after maximum intensity z-projection, bar equals 10 μm. (**c**) Generation times of BY4741 cells cultured in SC + 100 μM methionine at the indicated concentrations of cycloheximide in the absence (grey boxes) or presence (black boxes) of 20 μM SeMet. The results are the mean and range of at least 2 experiments. (**d**) Effect of reducing growth rates on SeMet toxicity and protein aggregation. The fraction of cells containing at least one Hsp104-GFP focus (⚫) was determined from the images in panel b, by visual inspection of 150–200 cells in each condition. SeMet growth inhibition (◼) was calculated as the log_2_(T^SeMet^/T^cont^) value where T^SeMet^ and T^cont^ are the generation times, calculated for each cycloheximide concentrations in panel c, in the presence and absence of SeMet, respectively. Values were plotted against the generation time in the absence of SeMet (T^cont^). The results are the mean and range of at least 2 experiments.

Most of the null alleles strains displaying a SeMet-resistant phenotype are involved in the process of translation or regulation of translation. These deletion strains generally display growth rate defects^25^. As shown in Fig. 3a, 80% of the SeMet-resistant mutants grew more slowly than the parental strain in the absence of stress, suggesting that a decreased growth rate provides an advantage under SeMet stress. Therefore, we checked whether slowing the rate of protein synthesis reduced SeMet-induced protein aggregation and toxicity. Low cycloheximide concentrations (0 to 100 ng/mL) were used to decrease cellular growth rate by 25, 45 and 65%. Reduction of translation rates was accompanied by a reduction of toxicity as well as number of cells containing aggregates (Fig. 3b,c,d), suggesting that protein aggregation is the cause of SeMet toxicity.

### Protein aggregation is dependent on SeCys synthesis

**Figure 4:**
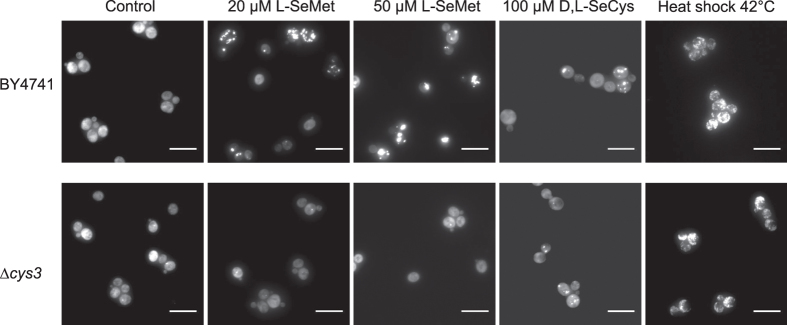
The presence of *CYS3* is required for SeMet-induced protein aggregation. Hsp104-GFP localization in BY4741 and BY4741-*∆cys3* cells after 1 h of exposure in SC + 100 μM methionine + 100 μM cysteine to 0, 20 μM and 50 μM SeMet, 100 μM D,L-SeCys in the presence of 1 mM TCEP or after 1 h heat shock at 42 °C was monitored in living cells by fluorescence microscopy. Representative images obtained after maximum intensity z-projection, bar equals 10 μm.

Misincorporation of SeMet into proteins in place of methionine is not believed to induce major cytotoxicity^22^. By constrast, because SeCys is much more reactive than its sulfur-analogue, misincorporated SeCys is susceptible to form adducts with thiols or selenols generating misfolded aggregation-prone proteins. Therefore, we investigated protein aggregation in a ∆*cys3* mutant strain in which SeCys cannot be formed from SeMet. To support cell growth, this analysis was performed in the additional presence of 100 μM cysteine. In the absence of SeCys synthesis, Hsp104-GFP distribution remained diffuse in the cytoplasm up to 50 μM SeMet in the medium (Fig. 4), a SeMet concentration in which foci were detectable in 80% of wild-type cells. Direct exposure to SeCys (100 μM D,L-SeCys) induced aggregation in 35% of the cells in both strains. Upon heat shock, similar aggregate formation was observed in the ∆*cys3* and wild-type cells. These results, together with the requirement for *CYS3* in SeMet toxicity, indicate that metabolization of SeMet to SeCys is necessary to generate protein aggregation and its associated toxicity.

### Selenoamino-acids incorporation in polypeptide chains

Unspecific insertion of SeCys in proteins has already been observed in yeast cells grown in the presence of selenite^12^. To determine whether SeCys can be incorporated into proteins when yeast cells are grown in the presence of SeMet, we used a strategy based on mass spectrometry. Yeast cells, expressing a tagged-version of Elongation Factor 1 (EF1-α), were grown in SC medium containing 20 μM SeMet and 100 μM methionine. EF1-α was chosen because it is a soluble medium size protein containing a significant number of cysteinyl residues and an equivalent number of methioninyl residues (7 and 8, respectively). After purification and proteolytic (Asp-N) digestion, the peptides were analyzed by LC-MS/MS and selenium substitution for sulfur was searched for and quantified. Protein sequence coverage was 86% with information for all of the sulfur-containing amino acids except the N-terminal methionine. Supplementary Figures 4, 5 and 6 show the ESI-MS/MS analyses and extracted ion chromatograms (XIC) of selected selenium-containing peptides (peptide 426–439 containing SeMet, peptides 110–117 and 360–370 containing SeCys). Table 2 summarizes the results of the mass spectrometric analysis. Insertion of a SeCys residue in replacement of cysteine was unambiguously identified at 3 positions (Cys111, Cys335, Cys361). The SeCys/Cys substitution ratio was 4.9 ± 3.5%. Selenium substitution for sulfur was found in 6 out of 7 methioninyl residues with an average Se/S substitution of 17.1 ± 5.3%, very close to the extracellular ratio of SeMet/Met.

Overall, these results suggest that SeMet-induced protein aggregation results from the metabolization of SeMet to SeCys and incorporation of SeCys in the place of cysteine in the course of translation.

## Discussion

**Figure 5:**
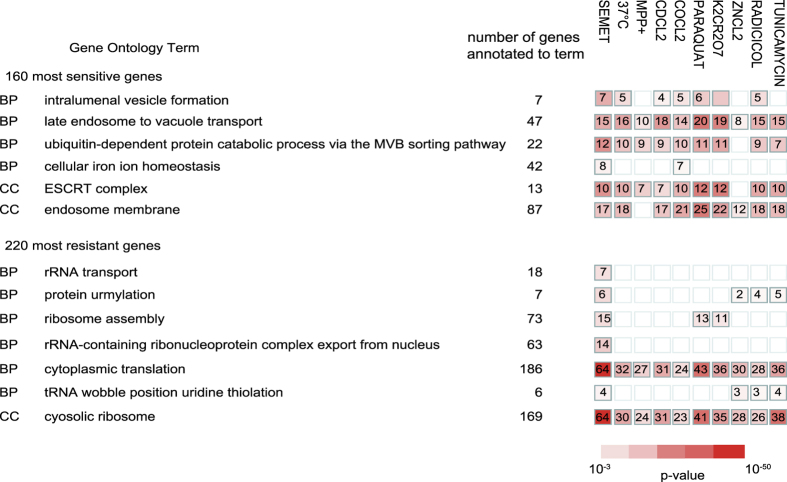
Compounds sharing significantly enriched GO terms with SeMet simultaneously in the sensitive and resistant datasets. The 160 most sensitive, or 220 most resistant, genes corresponding to SeMet (this study), compounds n°3 (37 °C), 180 (CdCl_2_), 181 (ZnCl_2_), 182 (CoCl_2_), 184 (K_2_Cr_2_O_7_), 374 (Paraquat), 375 (MPP+) in ref. 28 and compounds n°828 (Radicicol), and 4177 (Tunicamycin) in ref. 29 were analyzed for simultaneous functional enrichment with the g:Profiler multiple gene lists tool (http://biit.cs.ut.ee/gprofiler/gcocoa.cgi). Only processes significantly enriched (p-value < 0.001) in the SeMet screen are represented. The number of genes annotated to the GO term in the input lists is indicated with a color code corresponding to the p-value of the enrichment. Abbreviations: BP, Biological process; CC, Cellular component.

Chemogenomic studies in yeast have been previously applied to hundreds of growth inhibitory chemicals with the aim of uncovering their mechanisms of action. These studies have helped to identify biological functions involved in the toxicity of several compounds including metals, pesticides and pharmaceutical drugs and have also proven useful to shed light on human diseases^26^,^27^. In this study, a *S. cerevisiae* deletion collection was employed to better understand the molecular mechanisms underlying SeMet toxicity. The spectacular enrichment of functions related to protein metabolic processes, including biosynthesis, folding and degradation, in both the sensitive and resistant datasets suggests that SeMet causes a proteotoxic stress. We searched the yeast fitness databases generated in large-scale studies by Giaever *et al*.^28^ and Hoepfner *et al*.^29^ for chemical genetic profiles with similarities to that of SeMet, potentially reflecting common biological targets or mode-of-action^30^,^31^. Figure 5 shows a list of several stresses that share enriched GO terms with SeMet in both the sensitive and resistant datasets suggesting that all these stresses might affect protein functions. Indeed, exposure to elevated temperatures is known to destabilize protein structure and increase aggregation of improperly folded proteins. Several metals, including cadmium, cobalt and chromium, trigger oxidative protein damage and aggregation in yeast^32^,^33^. In addition, mRNA mistranslation due to sulfur starvation was shown to be a cause of hexavalent chromium toxicity^34^,^35^. Paraquat and 1-methyl-4-phenylpyridinium (MPP+) are environmental toxicants associated with an increased risk of developing Parkinson’s disease, a neurodegenerative pathology characterized by an accumulation of insoluble protein deposits. Exposure of dopaminergic cells to paraquat or MPP+ was shown to decrease protein ubiquitination, leading to a dysfunction of protein degradation pathways^36^. The molecular target of radicicol is the heat-shock protein Hsp90p, the inhibition of which affects protein quality control^37^. Lastly, tunicamycin disrupts protein folding in the endoplasmic reticulum (ER) and activates the unfolded protein response^38^.

In this study, we demonstrate that SeMet induces protein aggregation in a concentration-dependent manner by a mechanism that requires *de novo* protein synthesis. We observed a direct correlation between SeMet toxicity and protein aggregation. Slowing down translation, either by deletion of ribosomal protein genes or by using cycloheximide, improves SeMet resistance, suggesting that toxicity results from an impairment of cellular protein homeostasis that overwhelms cellular defenses against aggregated proteins. Reduction of the rate of translation elongation may improve the fidelity of translation and/or reduce the burden of the protein quality control machinery, both resulting in improved proteostasis^39^. Because nearly complete replacement of methionine by SeMet is tolerated by yeast cells without measurable adverse effects^22^,^40^, it is unlikely that SeMet mistranslation causes massive aggregation. Several lines of evidence suggest that SeCys misincorporation in nascent proteins could be responsible for protein aggregation. Fistly, we recently showed that the toxicity of SeMet involved its metabolization to transsulfuration pathway amino acids^19^. Secondly, as observed earlier and again here, toxicity is drastically reduced in a *∆cys3* mutant. Concomitantly, protein aggregation is not observed in this mutant exposed to SeMet. In contrast, exposure to SeCys of wild-type as well as *∆cys3* cells results in visible aggregation. Lastly, using cells grown in the presence of SeMet, we show that SeCys can be translationally incorporated in a reporter polypeptide. Random replacement of cysteine by the more reactive SeCys is likely to alter protein structure and induce misfolding by formation of non-native intermolecular or intramolecular selenylsulfide or diselenide bridges resulting in the formation of insoluble protein adducts. In addition, production of superoxide radicals during auto-oxidation of SeCys^19^ may also catalyze the oxidation of amino acid side chains^41^ contributing to aggregation of oxidized proteins. In particular, methioninyl residues are readily oxidized to methionine sulfoxide by ROS. Deletion of *MXR2*, one of the two genes encoding methionine sulfoxide reductase, results in a moderately sensitive phenotype (see Supplementary Table S1), suggesting that oxidation of methionine may contribute to SeMet toxicity. The hypersensitive phenotype of the *sod1* and *sod2* deletion mutants is also in agreement with an involvement of superoxides in the mechanism of protein aggregation.

To our knowledge, this is the first time that the molecular basis of SeMet toxicity is attributed to protein aggregation resulting from SeCys misincorporation. Examples of non-protein amino acids that can be incorporated into proteins and cause protein aggregation are already known. They include azetidine-2-carboxylic acid^42^, a proline analogue, or β-N-methylamino-L-alanine, the consumption of which has been associated with high incidence of amyotrophic lateral sclerosis^43^. Translational errors inducing protein misfolding have been associated with several pathologies including neurodegeneration^44^. For example, the mouse sticky mutation, which causes cerebellar Purkinje cell loss and ataxia, results from the substitution of serine for alanine due to a mutation in the editing domain of alanyl-tRNA synthetase^45^.

The mechanism of SeMet toxicity evidenced here in yeast may be significant in other organisms. In higher plants, exposure to SeMet is not expected to result in SeCys misincorporation due to the absence of the pathway for conversion of methionine to cysteine. However, in plants exposed to inorganic selenium, the hypothesis that cysteine nonspecific replacement by SeCys partly accounts for selenium toxicity has already been discussed^46^. In particular, Se-hyperaccumulator plants with high tolerance to selenium were shown to contain a SeCys methyltransferase, which methylates SeCys and prevents its incorporation into proteins^47^. Minimizing the misincorporation of SeCys by introduction of a SeCys methyltransferase gene has also been an effective strategy to increase Se tolerance in plants^48^,^49^. In addition, the ubiquitin-proteasome pathway, which we observe here to be important for SeMet tolerance in *S. cerevisiae* (Table 1), was recently implicated in SeCys toxicity in the plant *Brassica napus*^50^. In cultured human cells, an increase of protein-bound selenium and the induction of an ER stress response were observed upon selenocystine treatment^51^, leading the authors to suggest that replacement of cysteine by SeCys in polypeptides triggered an accumulation of misfolded proteins. In this study, GO analysis did not reveal enrichment for components of the ER-associated protein degradation pathway or for ER-related unfolded protein response in the sensitive or resistant subsets. However, the similarity between the genetic profiles of SeMet and tunicamycin, a known ER stress inducer, indicates that SeMet may also induce an ER stress in yeast.

Our present work using yeast cells establishes that SeMet toxicity is mediated by protein aggregation. Down-regulation of protein synthesis rates improved resistance to SeMet. Reciprocally, fast growing cells were more sensitive to SeMet addition. Our findings bring new insights into the molecular mechanisms underlying selenoamino acids toxicity. They may help in designing protocols for clinical studies aiming to compare the health benefits versus negative consequences of supplementation with different forms of selenium^52^.

## Methods

### Strains and media

The *S. cerevisiae* strains used in this study are derived from strain BY4742 (*MAT*α *his3∆1 leu2∆ lys2∆0 ura3∆0*) or BY4741 (*MAT*a *his3∆1 leu2∆ met15∆0 ura3∆0*). The parental strains and all the single mutants were obtained from Euroscarf. The BY4741 strain containing a GFP-tagged Hsp104 was purchased from ThermoFisher Scientific. GFP tagging of Hsp104 in yeast mutant strains was performed by PCR-mediated homologous recombination, and correct integrations were checked by PCR. Standard Synthetic Defined (SD) medium contained 0.67% (w/v) yeast nitrogen base (Difco), 2% (w/v) glucose and 50 mg/l of histidine, leucine, lysine and uracil and was buffered at pH 6.0 by the addition of 50 mM MES-NaOH. Standard Synthetic Complete (SC) medium contained 0.67% (w/v) yeast nitrogen base (Difco), 2% (w/v) glucose and 80 mg/l of adenine, uracil and all amino acids (160 mg/ml leucine) except methionine and cysteine and was buffered at pH 6.0 by the addition of 50 mM MES-NaOH. Media were supplemented in methionine and cysteine as indicated in the legends to the figures. All the amino acids were supplied as the L-enantiomers except SeCys which was the D,L-mixture. Because of its extreme sensitivity to oxidation, SeCys was prepared extemporaneously by reducing 2.5 mM selenocystine (Sigma) with 5 mM Tris (2-carboxyethyl) phosphine (TCEP) in 50 mM MES-NaOH, pH 6.0.

The pool of 4,885 *S. cerevisiae* mutants from the systematic deletion collection made in strain BY4741^15^,^28^ was mixed with 979 barcoded DAmP strains of essential genes^53^. Pooled strains were pregrown for 5 generations in SC medium containing 100 μM methionine and 100 μM cysteine. Growth in the same medium supplemented with 0, 12 or 20 μM SeMet was started at an OD_650_ of 0.002. All three cultures were grown aerobically at 30 °C, under shaking for 10 generations before harvesting cells.

For individual growth rate analysis, selected mutants were inoculated in the indicated medium supplemented with 0 or 20 μM SeMet at an OD_650_ of 0.0025 and cell growth at 30 °C was monitored by measuring the OD_650_ at various times during 24 h. The generation times were derived from the fit of experimental data to an exponential curve.

### Analysis of microarray data

Microarray results were obtained using Agilent custom slides as described^54^. Briefly, DNA was extracted from pools of mutants at the start of the experiment (t = 0) and after growth (10 generations) in the presence or absence of SeMet, and barcode sequences were amplified with fluorescent-labelled oligonucleotides. Microarrays were scanned in a Genepix 4000B scanner (Molecular Devices) and the images were analyzed using GenePix Pro. The spot values were normalized using R scripts to report the relative fitness scores defined as the log_2_(treated/untreated) values. Relative growth fitness score of the mutant strains in the absence of SeMet was defined as the log_2_(untreated/t = 0) values.

### Fluorescence microscopy

Yeast cells expressing Hsp104-GFP were grown at 30 °C to an OD_650_ of 0.5 in SC medium supplemented with methionine and cysteine as indicated in the legends, followed by exposure to SeMet, to SeCys, or transfer to 42 °C, for 1 h. Where indicated, cells were treated with cycloheximide at the indicated concentration for 1 h prior to SeMet addition. Cells were mounted on glass slides covered with a thin layer of 1% agarose. Differential interference contrast (DIC; Nomarski interference contrast) and fluorescence images were obtained at room temperature using a ZEISS Axio Observer equipped with a 40×, 1.4 NA oil immersion objective. 470 nm excitation at maximum available intensity (4 W cm^−2^) and a filter set 65 HE (EX BP 475/30, BS FT 495, EM BP550/100) were used for fluorescence imaging. The lateral resolution was estimated to be 180 nm. Images were captured with digital camera AxioCam MRm. Z-stacks of 15 to 25 images with 260 nm spacing were recorded. Maximum intensity z-projections were performed with ImageJ and the images were analyzed manually.

### Fluorescence spectroscopy

BY4741 cells expressing Hsp104-GFP (and a control untagged strain) were grown at 30 °C to an OD_650_ of 0.5 in SC + 100 μM methionine, followed by 2 h exposure to 12.5 and 20 μM SeMet, or transfer to 42 °C. Whole cell extracts were prepared in 50 mM Tris-HCl (pH 7.5), 10 mM MgCl_2_, 250 mM NaCl, 5% (v/v) glycerol, 10 mM 2-mercaptoethanol (2-ME), by vortexing cells at 4 °C for 10 × 30 sec in the presence of an equal volume of glass beads (500–750 μm). After centrifugation at 10000 × g for 10 min, the supernatant was recovered and the optical density at 280 nm was determined. GFP fluorescence was recorded at 508 nm in a Jasco FP-8300 spectrofluorometer using an excitation wavelength of 487 nm (bandwidth 2.5 nm).

### Identification of sulfur substitution in EF1-α

His-tagged EF1-α was purified and digested as described in Supplementary information. The nano LC–MS/MS analyses were performed as described^55^. Protein identification was performed using the Mascot database search engine (Matrix Science, London, UK) against the EF1-α-tag sequence (Supplementary Figure 4A) with semi-Asp-N Ambic specificity and 4 missed cleavages. Variable modifications included substitutions of cysteine and methionine by SeCys and SeMet, respectively, carbamidomethylation of cysteine and SeCys, oxidation of methionine and SeMet and conversion of SeCys to dehydroalanine. Peptide and fragment tolerance were respectively set at 10 ppm and 0.05 Da. Only peptides with Mascot ion scores above identity threshold (25) at less than 1% FDR (false discovery rate) were considered. MS extracted-ion chromatograms (XIC) of ^80^Se and ^32^S isotope peaks were generated using PeakView software (ABSciex). For relative quantification of peptides and selenized counterparts, incorporation rates were calculated as described^12^. Briefly, XIC peak areas were corrected by respective isotopic abundance and all forms described in variable modifications section were taken into account in the calculation of the substitution rates.

## Additional Information

**Accession codes:** The raw microarray data has been deposited in the NCBI GEO database under accession n° GSE84340.

**How to cite this article**: Plateau, P. *et al*. Exposure to selenomethionine causes selenocysteine misincorporation and protein aggregation in *Saccharomyces cerevisiae. Sci. Rep.* **7**, 44761; doi: 10.1038/srep44761 (2017).

**Publisher's note:** Springer Nature remains neutral with regard to jurisdictional claims in published maps and institutional affiliations.

## Supplementary Material

Supplementary Information

Supplementary Table 1

## Figures and Tables

**Table 1:** List of sensitive and resistant genes and associated GO terms.

**Significant SeMet sensitive mutants and associated GO terms**
**Ubiquitin-dependent protein catabolic process**
*UBP6, UBP14, RPT1, RPT2, RPT3, RPT4, RPT5, RPT6, DOA1, UBR2, HRT1, MUB1*
**- via the multivesicular body sorting pathway**
*STP22, DOA4, GGA2, VPS25, DID4, VPS24, SNF7, SRN2, VPS36, VPS20, SNF8, BRO1*
**Endosome membrane**
*ATG15, VPS4, VPS27, VPS38, DID2*
**Superoxide metabolic process**
*SOD1, SOD2*
**Sulfur compound metabolic process**
*MET13, ADE3, GSH2*
**Prefoldin complex**
*PAC10, GIM5, YKE2*
**Significant SeMet resistant mutants and associated GO terms**
**tRNA processing**
*TRM1, LOS1, MOD5, STP1, CGI121, PUS7*
**- tRNA wobble uridine modification**
*ELP2, ELP4, ELP6, NCS2, NCS6, UBA4, URM1, IKI3*
**Ribosome biogenesis**
*ECM1, SYO1, ARX1, RPA14, LOC1, CGR1, DBP3, SPT4, SLX9, TIF4631, PIH1, LRP1, RTC3, RPA34, MRT4, LTV1, FYV7, BUD20, HCR1, TSR2, TMA23, JJJ1, RPA49, NOP12, BUD21, PUS7, SRP40*
**Ribosome**
*RPL4A, RPS14A, RPP1A, RPL13A, RPP1B, RPP2B, RPS17B, RPL27B, RPL37B, RPL34A, RPS24A, RPL29, RPL24A, RPL7A, RPL28, RPL9A, RPS25A, RPL26B, RPL11B, RPL24B, RPS0A, RPL8A, RPL16A, RPL17B, RPL14A, RPS21A, RPL8B, RPS0B, RPL22A, RPL37A, RPS28B, RPS29A, RPL31B, RPS1A, RPL6B, RPS17A, RPS1B, RPL13B, RPL36A, RPS10B, RPL9B, RPL16B, RPS19B, RPS19A, RPS7A, RPL33B, RPL21B*
**Sulfur compound metabolic process**
*SAM1, SAM2, MUP1, CYS3, MET12, MET31*
**Lsm1-7-Pat1 complex**
*LSM1, LSM6, LSM7, PAT1*
**Regulation of transcription**
**- RNA polymerase II mediator complex**
*MED2, SSN2, SSN3, SSN8, GAL11, PGD1*
**- SAGA complex**
*GCN5, NGG1, ADA2, SGF29, CHD1*

**Table 2:**
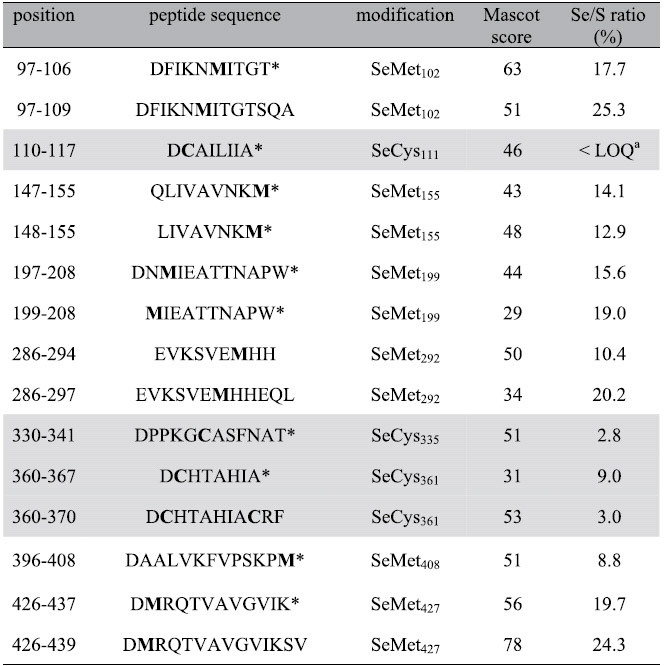
Ratio of Se/S substitution in EF1-α sulfur-containing peptides.

Cysteine-containing peptides are in light grey boxes. Sulfur-containing residues are in bold.

*Peptides resulting from specific cleavage only at one terminus.

^a^XIC peak areas for peptides 110–117 were above limit of detection (LOD) but lower than limit of quantification defined as LOQ = 3 X LOD.
