# Supplementary material for: Exposure to selenomethionine causes selenocysteine misincorporation and protein aggregation in Saccharomyces cerevisiae
Source: Sci Rep. 2017 Mar 17;7:44761. doi: 10.1038/srep44761 (PMC5355996; doi:10.1038/srep44761)
Supplement: Supplementary Information [file srep44761-s1.pdf]

Supplementary Information for "Exposure to selenomethionine causes selenocysteine misincorporation and protein aggregation in Saccharomyces cerevisiae" by P. Plateau, C. Saveanu, R. Lestini, M. Dauplais, L. Decourty, A. Jacquier, S. Blanquet and M. Lazard

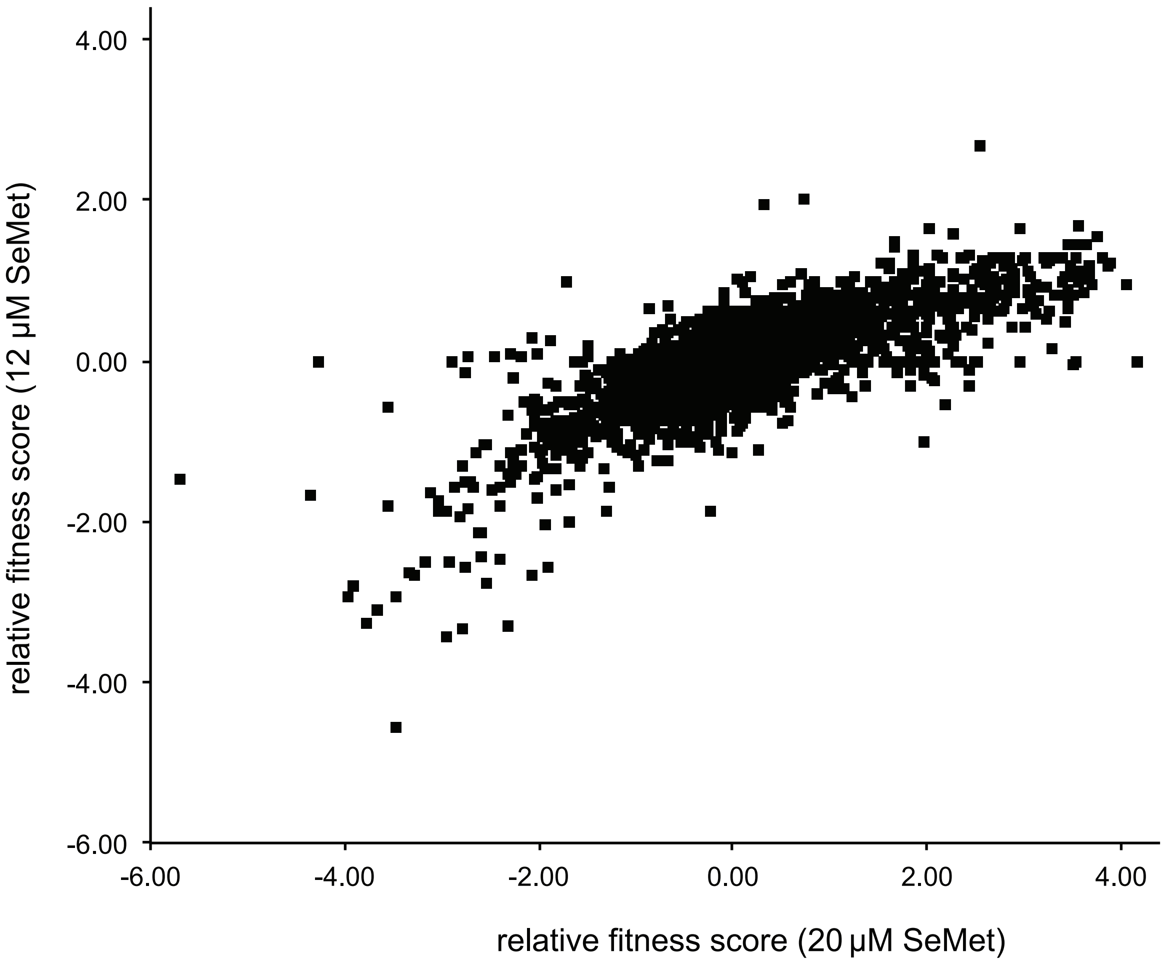

Supplementary Figure 1.
Relative fitness scores of the deletion strains grown in the presence of 12 µM SeMet (in ordinate) or 20 µM SeMet (in abscissa).

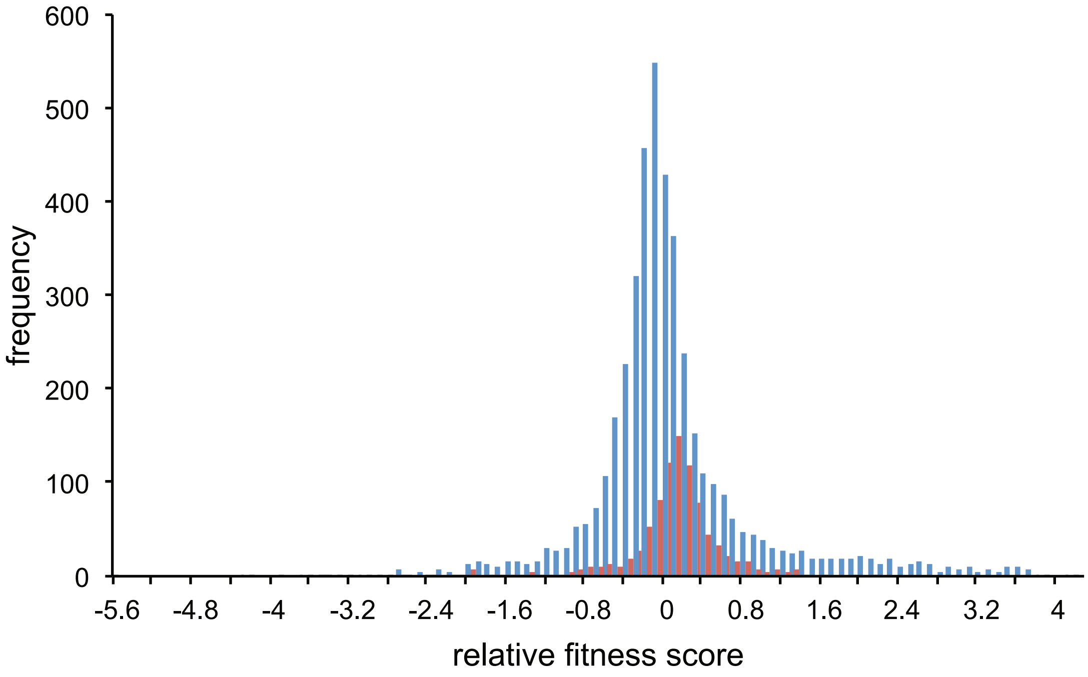

Supplementary Figure 2.
Distribution of the relative fitness scores among the deletion strains (in blue) and DAmP strains (in red) grown in the presence of 20 µM SeMet. Size of bins = 0.1

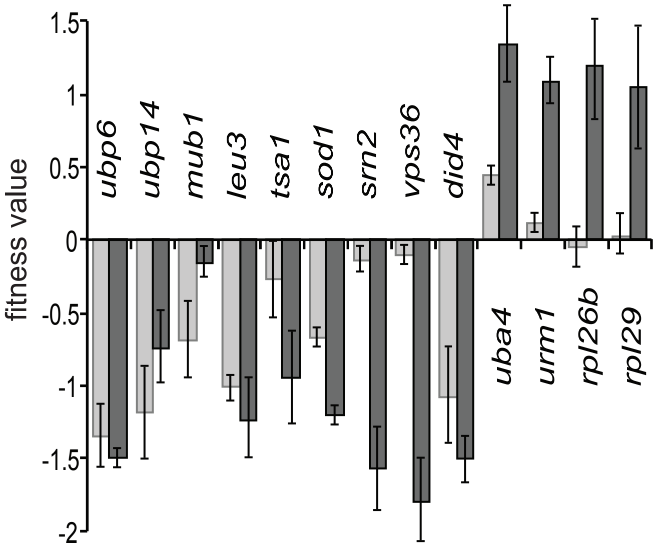

Supplementary Figure 3. Fitness value of selected mutants relative to the BY4742 parental strain. Exponential growth rates of wild-type strain and indicated haploid mutants were measured in the absence or the presence of 20 μM SeMet in SD + 100 μM methionine (light grey boxes) or SC + 100 μM methionine (dark grey boxes) medium. To compare with values obtained in the genomic screen (growth for 10 generations), fitness values were calculated according to the following equation $10 \times (T_{w.t}^{SeMet} / T_{mutant}^{SeMet} - T_{w.t} / T_{mutant})$ where T is the generation time. The value indicated in the figure corresponds to the average and range of duplicate experiments. Generation times measured for the wild-type in SD + 100 μM methionine medium were 116 and 178 min in the presence of 0 and 20 μM SeMet, respectively. They were 98 and 158 min in SC + 100 μM methionine medium.

A

1 **M**GKEKSHINV VVIGHVDSGK STTTGHLIYK CGGIDKRTIE KFEKEAAELG KGSFKYAWVL DKLKAERERG

71 ITIDIALWKF ETPKYQVTVI DAPGHR**DFIK** **NMITGTSQAD** **CAILIIA**GGV GEFEAGISKD GQTREHALLA

141 FTLGVR**QLIV** **AVNKM**DSVKW DESRFQEIVK ETSNFIKKVG YNPKTVPFVP ISGWNG**DNMI** **EATTNAPW**YK

211 GWEKETKAGV VKGKTLLEAI DAIEQPSRPT DKPLRLPLQD VYKIGGIGTV PVGRVETGVI KPGMVVTFAP

281 AGVTT**EVKSV** **EMHH**EQLEQG VPGDNVGFNV KNVSVKEIRR GNVCGDAKN**D** **PPKGCASFNA** **T**VIVLNHPGQ

351 ISAGYSPVL**D** **CHTAHIACRF** DELLEKNDRR SGKKLEDHPK FLKSG**DAALV** **KFVPSKPM**CV EAFSEYPPLG

421 RFAVR**DMRQT** **VAVGVIKSV**D KTEKAAKVTK AAQKAAKKHH HHHHGRIFYP YDVPDYAGLE VLFQGPGPSA

491 VDNKFNKEQQ NAFYEILHLP NLNEEQRNAF IQSLKDDPSQ SANLLAEAKK LNDAQAPKVD NKFNKEQQNA

561 FYEILHLPNL NEEQRNAFIQ SLKDDPSQSA NLLAEAKKLN DAQAPKVDAN HQ

B

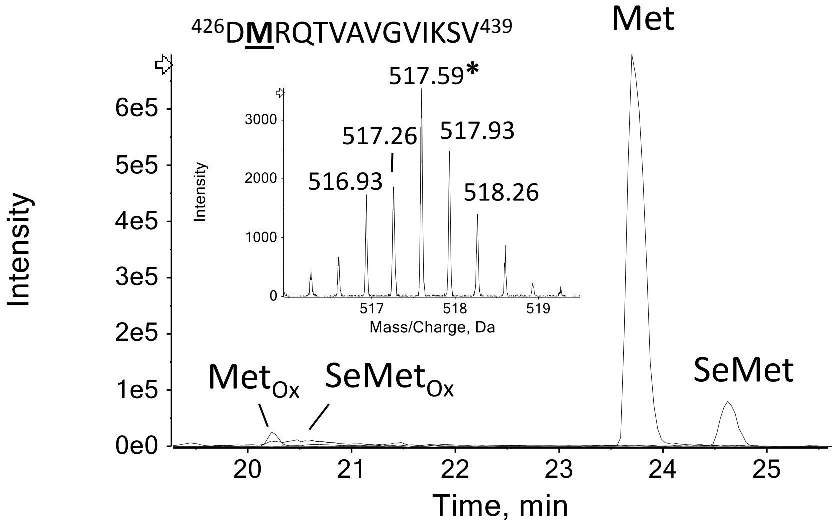

C

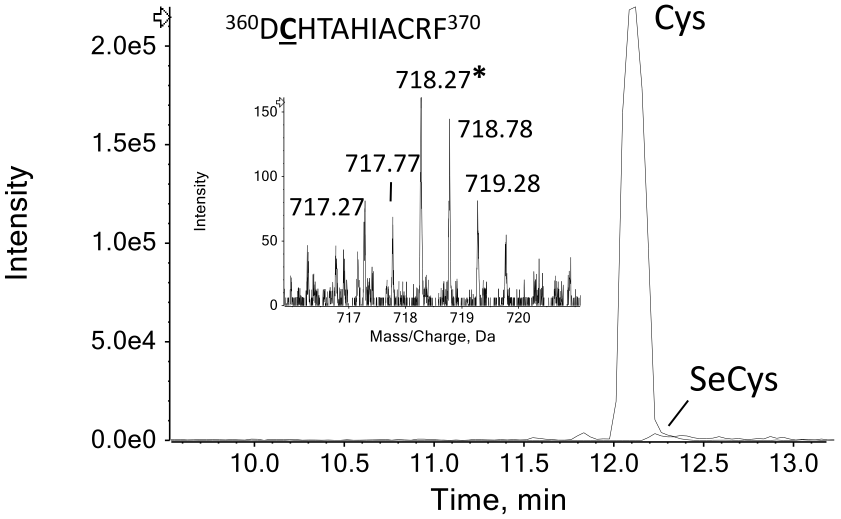

Supplementary Figure 4. ESI-MS analysis of purified EF1-α

(A) Amino acid sequence of tagged EF1-α. Selenium-containing peptides identified by MS/MS analysis are shown in bold. Methioninyl and cysteinyl residues are boxed in grey. (B) MS extracted-ion chromatogram (XIC) of the 426-439 peptide and its selenized counterpart. Panel in insert shows the mass spectrum of the triply charged selenized peptide. Peak labeled with asterisk corresponds to the ^{80}Se isotope peak. (C) MS extracted-ion chromatogram (XIC) of the 360-370 peptide and its selenized counterpart. Panel in insert shows the mass spectrum of the doubly charged selenized peptide. Peak labeled with asterisk corresponds to the ^{80}Se isotope peak.

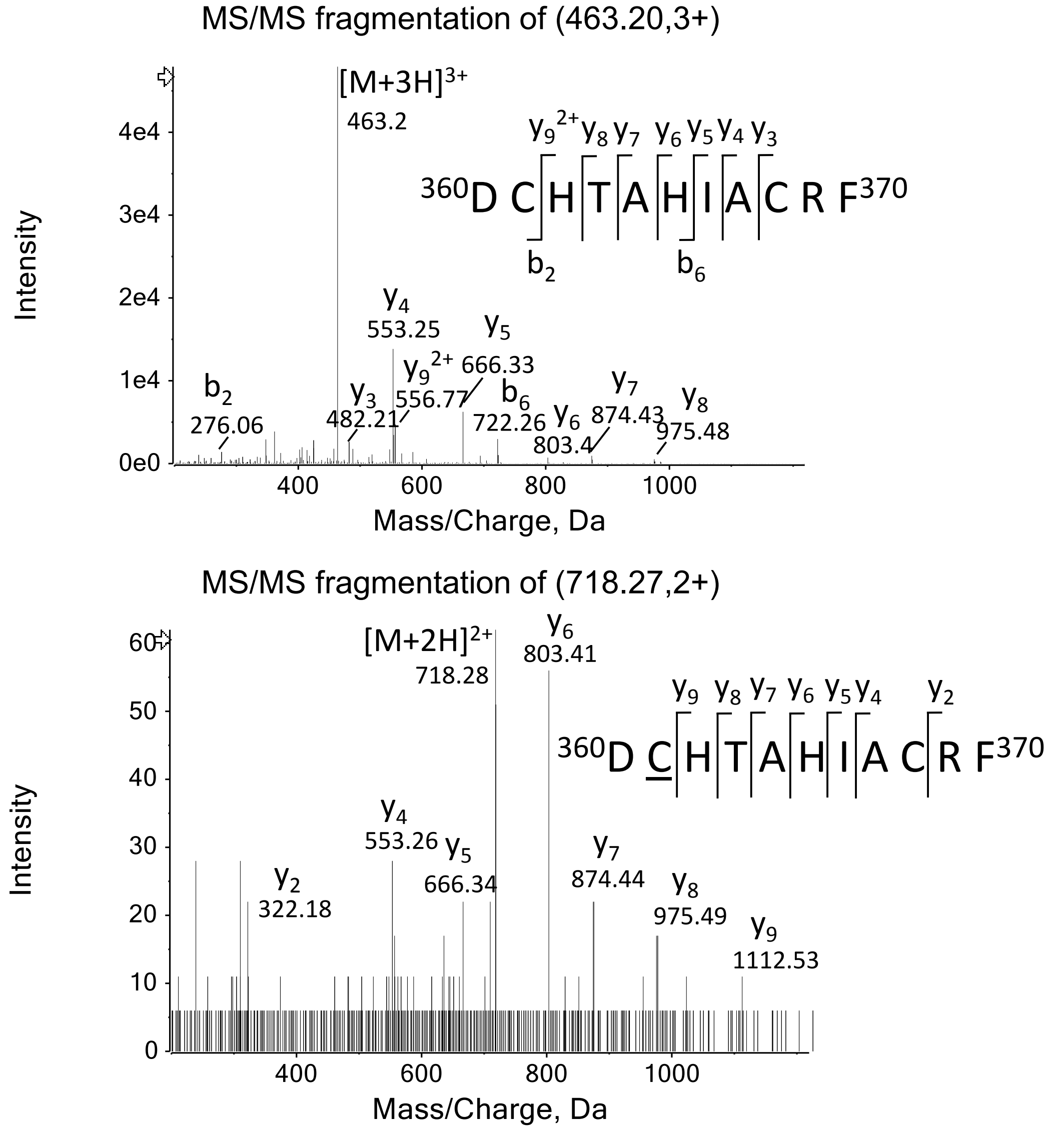

Supplementary Figure 5.

Comparison of the MS/MS fragmentation spectra of peptides 360-370 containing sulfur (upper panel) or selenium (lower panel). Identified fragments and their charge states are annotated on sequence. The difference in mass between the two precursor ions (47.94) indicates that the selenized 360-370 peptide contains only one selenium atom. Common fragments y_4 to y_9 in the two spectra indicate that this SeCys is located at position 361 (underlined on sequence).

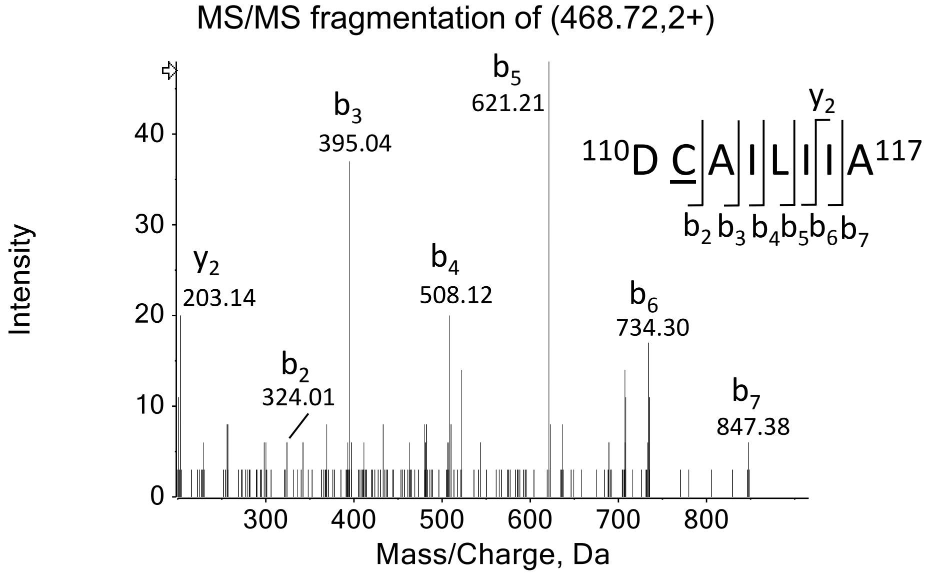

Supplementary Figure 6.
MS/MS fragmentation spectrum of peptide 110-117 containing selenium

Supplementary Table S1 (provided as a separate file). Global SeMet fitness profiling for all the 5241 strains with at least 2 independent results for the growth in the presence of 20 µM SeMet.

Supplementary Experimental Procedures

Purification and proteolytic digestion of EF1-α

The BY4741 strain containing *TEF2* expressed from the BG1805 plasmid (Dharmacon) was grown at 30°C in 1 L of SC medium (without uracil) containing 100 µM methionine to a OD_{650} of 0.8. Then, cells were harvested by centrifugation and resuspended in the same volume of medium containing 2% (w/v) galactose instead of glucose and 20 µM SeMet. After 30 h incubation at 30°C, cells (2.2 OD_{650}) were harvested by centrifugation, washed, and resuspended in a 100 mM ammonium acetate buffer (pH 7.5) containing 200 mM NaCl, 10 mM imidazole, 5% glycerol (v/v), 2 mM 2-ME and protease inhibitors (1 mM phenylmethylsulfonyl fluoride, 0.5 µg /mL aprotinin, 0.5 µg /mL antipain, 0.5 µg /mL chymostatin, 0.5 µg /mL leupeptin, 0.5 µg /mL pepstatin A, 100 µg /mL benzamidine, 1 µg /mL *o*-phenantroline and 6 µg /mL ovomucoid). Cells were lyzed by vortexing at 4°C for 10 × 30 sec in the presence of an equal volume of glass beads. After centrifugation at 10000 × g for 10 min, the supernatant was adjusted to 5 ml, mixed with 1 ml of TALON resin (Clonetech) and incubated for 1 h at 4°C. After washing with 15 volumes of 100 mM ammonium acetate (pH 7.5), 10 mM imidazole and 2 mM 2-ME, elution was performed in the same buffer containing 125 mM imidazole. Thiols and selenols were reduced with 20 mM dithiothreitol for 30 min followed by alkylation with 50 mM iodoacetamide for 1 h. After concentration to 1 mg/mL, the sample was extensively dialyzed against 100 mM ammonium acetate (pH 7.5). Proteolytic digestion was performed overnight at 37°C after addition of 200 ng of Asp-N (Promega, Madison, WI, USA) to 5 µg protein sample diluted in 25 mM NH_4HCO_3. Finally, 0.5 µg Asp-N-generated peptides were diluted in 0.1 % (v/v) trifluoroacetic acid and 5% (v/v) acetonitrile and analyzed by LC-MS/MS.
